# Lung Microenvironment Among Patients with Nontuberculous Mycobacterial Pulmonary Disease by Metagenomic Sequencing Technique

**DOI:** 10.3390/biomedicines13040818

**Published:** 2025-03-28

**Authors:** Le Qin, Yu Chen, Sichun Luan, Xiaoyu Yin, Jue Pan, Leilei Wang, Yumeng Yao, Chunmei Zhou, Rong Bao, Jiajin Shen, Qing Miao, Bijie Hu

**Affiliations:** 1 Department of Infectious Diseases, Zhongshan Hospital, Fudan University, Shanghai 200032, China; 18301050249@fudan.edu.cn (L.Q.); 22111210019@m.fudan.edu.cn (Y.C.); luan.sichun@zs-hospital.sh.cn (S.L.); 17301020037@fudan.edu.cn (X.Y.); pan.jue@zs-hospital.sh.cn (J.P.); wang.leilei@zs-hospital.sh.cn (L.W.); yao.yumeng@zs-hospital.sh.cn (Y.Y.); 2 Shanghai Institute of Infectious Disease and Biosecurity, Zhongshan Hospital, Fudan University, Shanghai 200032, China; 3 Department of Laboratory Medicine, Zhongshan Hospital, Fudan University, Shanghai 200032, China; zhou.chunmei@zs-hospital.sh.cn (C.Z.); bao.rong@zs-hospital.sh.cn (R.B.); shen.jiajin@zs-hospital.sh.cn (J.S.)

**Keywords:** nontuberculous mycobacterial pulmonary disease, next-generation sequencing, lung, microbiome, immunosuppression

## Abstract

**Background**: Nontuberculous mycobacterial pulmonary disease (NTM-PD) is an increasingly prevalent chronic infection, where the host immune status plays a crucial role in disease susceptibility and progression. The complex pulmonary microenvironment, characterized by diverse microbial communities and host immune interactions, exhibits distinct features that may be fundamentally altered by the patient’s underlying immune state. **Methods**: A total of 111 sputum specimens and 64 bronchoalveolar lavage fluid (BALF) specimens were collected from 143 patients diagnosed with NTM-PD under different immune states. Metagenomic sequencing was performed on these specimens to characterize and compare the pulmonary microenvironmental features among NTM-PD patients with a distinct immune status through comprehensive bioinformatic analyses. **Results**: The immunosuppressed group exhibited a lower α-diversity in sputum specimens (*p* < 0.05). Principal Coordinates Analysis (PCoA) of β-diversity for sputum and BALF specimens revealed significant differences between the groups (*p* < 0.05). Linear discriminant analysis Effect Size (LEfSe) analysis identified species enriched in the immunosuppressed group. A co-occurrence network analysis indicated that the immunosuppressed group had more structured and actively connected networks compared to the control group. The Mantel test confirmed that the abundance of these species enriched was associated with clinical immune–inflammation-related indicators in patients. **Conclusions**: Our study reveals the pulmonary microenvironment in immunosuppressed patients with NTM-PD. Further work is required to explore the two-way relationship between micro-organisms and immune and inflammatory responses, with the influence on patient outcomes.

## 1. Introduction

In recent years, a significant upward trend in Nontuberculosis *Mycobacterium* (NTM) infection incidence has been observed globally, where immunosuppressed patients are showing markedly increased susceptibility to these infections due to their impaired host defense mechanisms [1,2]. NTMs comprise over 190 species distinct from the *Mycobacterium tuberculosis* complex and *Mycobacterium leprae* complex, with varying degrees of virulence that can cause pulmonary and extrapulmonary diseases [3]. As NTM primarily manifests as pulmonary infections, NTM pulmonary disease (NTM-PD) has emerged as an increasingly critical public health concern [4,5]. The pulmonary microbiome is considered a crucial environmental factor in NTM infection onset, potentially influencing the process through pro-inflammatory responses, microbial metabolites, and energy balance [6,7,8]. From a biological perspective, various inflammatory factors [9,10,11], such as IL-6 and TNF-α, have been identified as participants in the NTM infection process. Intriguingly, no significant correlation has been observed between NTM abundance and the upregulation of these inflammatory markers [12], suggesting the involvement of commensal bacteria within the pulmonary microbiome in modulating the inflammatory response to NTM-PD.

Evidence indicates that immunosuppressed NTM-PD patients present with more complex clinical manifestations and poorer outcomes than immunocompetent individuals. Chai et al. [13] demonstrated that immune immunosuppression in NTM-PD correlates with an increased frequency of fever, higher rates of disseminated infection, and a significantly greater risk of clinical exacerbations—highlighting the impact of host immunity on disease progression and prognosis. However, studies on NTM infection mechanisms in immunosuppressed hosts remain relatively scarce. Therefore, this study aims to elucidate the alterations in microbial communities during the development of NTM pulmonary disease in immunosuppressed patients. This investigation is expected to provide novel insights into NTM pathogenesis and potentially furnish crucial evidence for the development of innovative therapeutic strategies.

## 2. Materials and Methods

### 2.1. Study Subjects

Our study collected specimens from 143 patients diagnosed with NTM at Zhongshan Hospital in Shanghai from 2019 to 2023. Among them, sputum specimens were obtained from 111 patients, and bronchoalveolar lavage fluid (BALF) specimens were collected from 64 patients. All patients included in the study exhibited respiratory symptoms, radiological evidence of bronchiectasis, or cavitary lesions with bronchiolitis, and had NTM isolated in cultures from two or more sputum specimens or at least one BALF specimen [14,15]. The criteria for defining immunosuppressed patients [16] were as follows: patients receiving long-term (>3 months) or high-dose (>0.5 mg/kg/day) corticosteroids or other immunosuppressive drugs, recipients of solid organ transplants, patients with solid tumors requiring chemotherapy within the past 5 years, patients with hematologic malignancies since diagnosis or treatment, or those with primary immunodeficiency. Meeting any of these criteria qualified a patient as immunosuppressed with NTM-PD. Immunocompetent NTM-PD patients were defined as those without primary immunodeficiency, malignant tumors, immune system disorders, a history of immunosuppressive therapy, blood system diseases, severe COPD, uncontrolled diabetes mellitus, or other factors known to compromise cellular immunity [17,18]. Baseline data for the patients were extracted from their admission histories. Recent antibiotic use was defined as the administration of any antibiotic agent (oral, intravenous, or inhaled) within 90 days prior to admission. A patient was considered to have a recent antibiotic history if they had used antibiotics for at least three consecutive days or had received a cumulative total of seven or more antibiotic doses within this period [19,20]. In this study, pulmonary co-infection was defined as the concurrent detection of other clinically relevant pathogens in the same respiratory specimens at the time of NTM pulmonary disease diagnosis, identified through standard microbiological methods (culture, PCR, etc.), accompanied by corresponding clinical manifestations and/or radiological evidence indicative of co-infection [21]. All participants provided written informed consent. This retrospective study was approved by the Ethical Committee of Zhongshan Hospital, Fudan University (approval number: B2020-411).

### 2.2. Metagenomic Sequencing

Patient specimens were immediately transferred to the hospital’s rapid Next-Generation Sequencing (NGS) platform upon collection. According to the specimens, we had to perform liquefaction treatment before extracting nucleic acids. The existing liquefaction schemes include the following methods: (i) standard N-acetyl-l-cysteine treatment and digestion with 2% NaOH (Sigma-Aldrich, St. Louis, MO, USA) and decontamination; (ii) NaOCl (Fisher Scientific, Waltham, MA, USA)liquefaction and sedimentation; (iii) chitin solution (Merck KGaA, Darmstadt, Germany) liquefaction; (iv) the use of sputum and an equal volume of phosphate buffer containing 1 g L^−1^ protease K (Thermo Fisher Scientific, Waltham, MA, USA)to liquefy the sputum; and (v) classical dithiothreitol (DTT) (Sigma-Aldrich, St. Louis, MO, USA) liquefaction, in which a chitin solution homogenizes the mucus sputum more quickly than the N-acetyl-l-cysteine and NaOCl methods. Then we used nucleic acid extraction kits (QIAGEN, Hilden, Germany) to extract DNA. The next step was library preparation and sequencing on the Illumina sequencing platform [22]. High-quality reads were aligned to the human reference genome (hg19) using the Burrows-Wheeler Aligner to eliminate human-derived sequences. The remaining sequences were subsequently mapped to the current RefSeq database for comparative analysis. This database, accessible from the National Center for Biotechnology Information (NCBI, https://www.ncbi.nlm.nih.gov/datasets/genome/, accessed on 15 October 2024), encompasses 3446 bacterial species (including 127 mycobacterial species), 4152 viral taxa, 206 fungal species, and 140 parasites associated with human diseases.

### 2.3. Immunological Phenotyping and Cytokine Analysis

Peripheral blood immune cell subsets and cytokine profiles were assessed using flow cytometry and multiplex immunoassay technology. Briefly, venous blood samples (5 mL) were collected from patients, with 2 mL anticoagulated with EDTA for immune cell analysis and 3 mL used for serum separation. For immune cell phenotyping, 100 μL of whole blood was incubated with fluorochrome-conjugated monoclonal antibodies against T-cell markers (CD3, CD4, CD8), a B-cell marker (CD19), and an NK-cell marker (CD56) for 20 min at room temperature in the dark, followed by red blood cell lysis and PBS washing^1^. Data were acquired using a FACSCanto II flow cytometer (BD Biosciences) and analyzed for the proportions of helper T-cells (Th, CD3+CD4+), cytotoxic T-cells (Ts, CD3+CD8+), and NK-cells (CD3-CD56+). For cytokine analysis, separated serum samples were simultaneously assayed for multiple inflammation-related cytokines including TNF-α, various interleukins (IL-1β, IL-2, IL-4, IL-6, IL-8, IL-10, IL-17), and interferon-γ (IFN-γ) using the Bio-Plex Pro™ multiplex cytokine detection system (Bio-Rad, Shanghai, China). Samples were loaded onto pretreated magnetic bead plates and processed following standard incubation, washing, and detection protocols, with results measured using the Bio-Plex 200 system [23,24]. All measurements were performed at least in triplicate.

### 2.4. Data Analysis

Statistical analyses were performed using various software packages. Independent specimen t-tests were conducted using SPSS version 26 to calculate means and significance for continuous variables, while chi-square tests were employed for non-continuous variables. Species occurring in less than 10% of specimens were excluded as confounding factors. α-diversity box plots were generated using the R packages vegan and ggplot2. The Shannon diversity index, which accounts for both the abundance and evenness of species present in a community, and the Chao1 richness estimator, which assesses total species richness, were calculated using the ’diversity’ function in the vegan package. Principal Coordinate Analysis (PCoA) was constructed based on Bray–Curtis distances. Wilcoxon signed-rank tests were utilized to calculate *p*-values for paired groups. To analyze inter-group differences, a Linear discriminant analysis Effect Size (LEfSe) was performed using Galaxy [25] and Wekemo Bioincloud (https://www.bioincloud.tech, accessed on 22 May 2024) [26]. Co-occurrence networks of NTM and environmental bacteria across different groups were analyzed using Gephi version 0.9.6 (https://gephi.org/, accessed on 15 August 2024). The R package linkET was employed for a correlation analysis between microbial abundance and clinical diversity (https://github.com/Hy4m/linkET, accessed on 17 November 2024) [27]. An Analysis of Covariance (ANCOVA) was used to evaluate the significance of differences in clinical characteristics, with gender, Chronic Obstructive Pulmonary Disease (COPD), and co-infection as covariates. A Spearman correlation analysis was conducted to calculate correlations between species and clinical indicators. Differences were considered significant when *p* < 0.05. Two-tailed Student’s *t*-tests were performed, with *p*-values adjusted using the Benjamini–Hochberg (BH) correction. The probability level for statistical tests was set at α = 0.05, adjusted by BH correction to allow for a maximum false discovery rate of 5% (q = 0.05). All data analyses were performed using IBM SPSS Statistics version 26 (IBM SPSS Inc., Chicago, IL, USA), R version 4.3.2 (R Foundation for Statistical Computing, Vienna, Austria), and Microsoft Excel (Microsoft Corporation, Redmond, Washington, DC, USA).

## 3. Results

### 3.1. Clinical Characteristics of Participants

Table 1 delineates the patient characteristics and clinical manifestations of NTM-infected individuals in both the immunocompetent and immunosuppressed groups. The data reveal significant disparities between the two cohorts. The immunosuppressed group exhibited a markedly lower proportion of female patients compared to the immunocompetent group (*p* = 0.02). Furthermore, the immunosuppressed cohort demonstrated higher rates of comorbid COPD (*p* = 0.04) and pulmonary co-infection-related conditions (*p* < 0.01). Although the frequency of recent antibiotic exposure was somewhat elevated in the immunosuppressed cohort compared to their immunocompetent counterparts (38% vs. 29%, respectively), this disparity did not reach statistical significance (*p* = 0.35). Interestingly, other clinical features, including comorbidities and radiological presentations, did not differ significantly between the two groups.

### 3.2. Microbiome Diversity Reduction Associated with Immunosuppression

**Figure 1:**
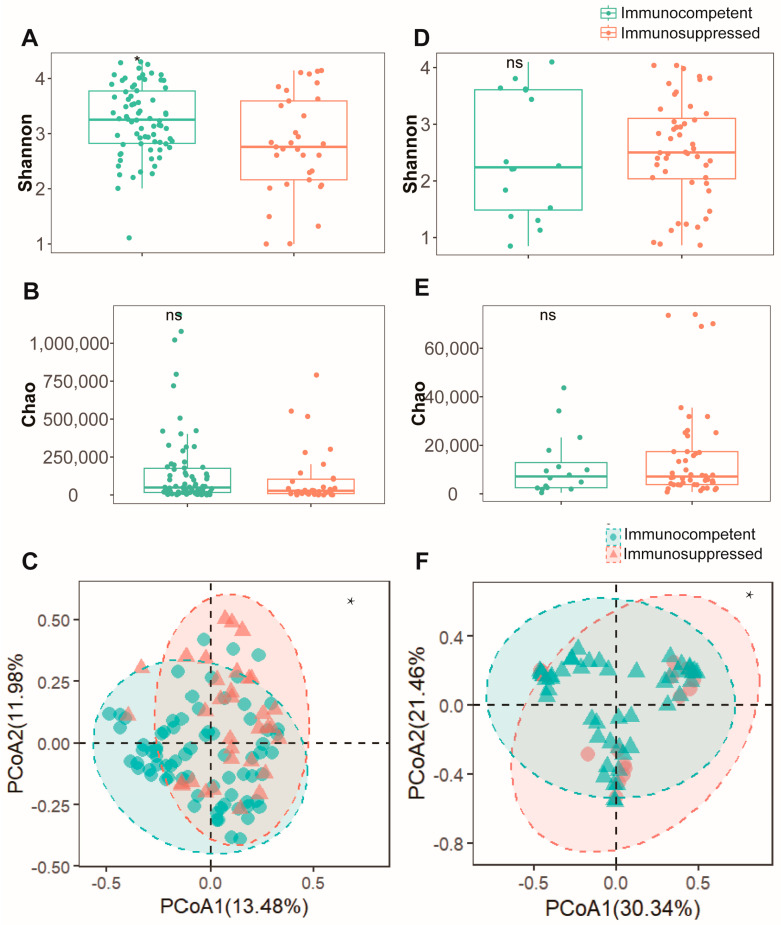
Microbial diversity analysis reveals comparable bacterial communities between immunocompetent and immunosuppressed patients in both sputum and BALF specimens. (**A**) Shannon diversity index and (**B**) Chao1 richness estimator showing alpha diversity metrics between immunocompetent (green) and immunosuppressed (orange) patients in sputum specimens. (**C**) Beta diversity analysis of sputum specimens visualized by PCoA based on Bray–Curtis dissimilarity matrix. Ellipses indicate 95% confidence intervals for each group. (**D**) Shannon diversity index and (**E**) Chao1 richness estimator demonstrating similar alpha diversity patterns between groups in BALF specimens. (**F**) Beta diversity analysis of BALF specimens visualized by PCoA between immunocompetent (green) and immunosuppressed (pink) patients. *: *p* < 0.05, ns: not significant (*p* > 0.05, Mann–Whitney U test).

To assess the differences in respiratory microenvironments between the two patient cohorts, both sputum and BALF specimens were analyzed. In sputum specimens, the immunosuppressed group exhibited a significantly lower Shannon index (Figure 1A) compared to the immunocompetent group (*p* < 0.05). However, no significant difference was observed in the Chao index between the groups (Figure 1B). A β-diversity analysis using PCoA revealed distinct microbial community patterns between the groups (Figure 1C). An analysis of BALF specimens showed no statistically significant difference in α-diversity either (Figure 1D,E). Nevertheless, the PCoA results indicated significant dissimilarities in microbial composition between these cohorts (Figure 1F). These findings suggest that alterations in the pulmonary microenvironment diversity of NTM-PD patients may be influenced by their immune status. Immunosuppressed patients typically demonstrate reduced microbial diversity in their pulmonary microenvironment.

### 3.3. Differential Microbial Analysis Associated with Immunosuppression

**Figure A1:**
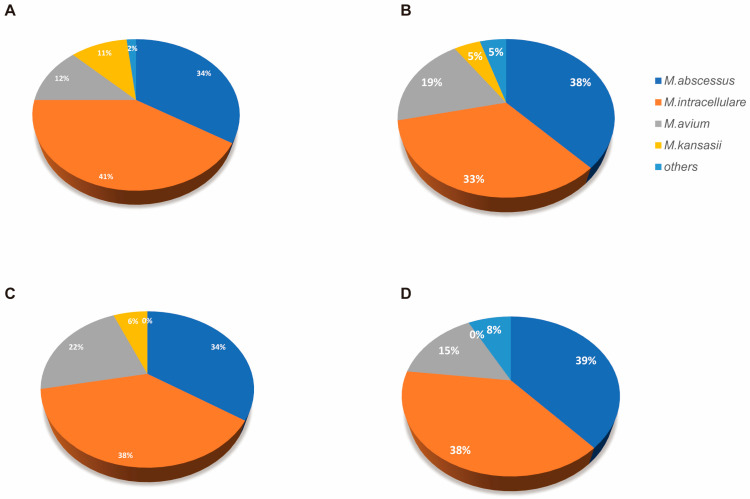
Identified NTM species percentages across different specimen types and immune status. Pie charts showing the relative proportions of different NTM species isolated from respiratory specimens. (**A**) Sputum samples from immunocompetent patients (*n* = 56). (**B**) Sputum samples from immunosuppressed patients (*n* = 21). (**C**) BALF specimens from immunocompetent patients (*n* = 32). (**D**) BALF specimens from immunosuppressed patients (*n* = 12).

An analysis of *Mycobacterium* distribution showed contrasting patterns across specimen types. In sputum specimens, immunosuppressed patients showed a lower abundance (0.75% vs. 1.60%, *p* = 0.01), with comparable detection rates between groups. Conversely, in BALF specimens, immunosuppressed patients exhibited a higher relative abundance (5.18% vs. 3.82%, *p* = 0.04), despite similar detection rates (Table 2). Our subsequent species identification of the detected *Mycobacterium* revealed that *M. intracellulare* and *M. abscessus* predominated in both populations. However, immunosuppressed patients harbored several rare NTM species that were absent in immunocompetent individuals (Figure A1).

**Figure 2:**
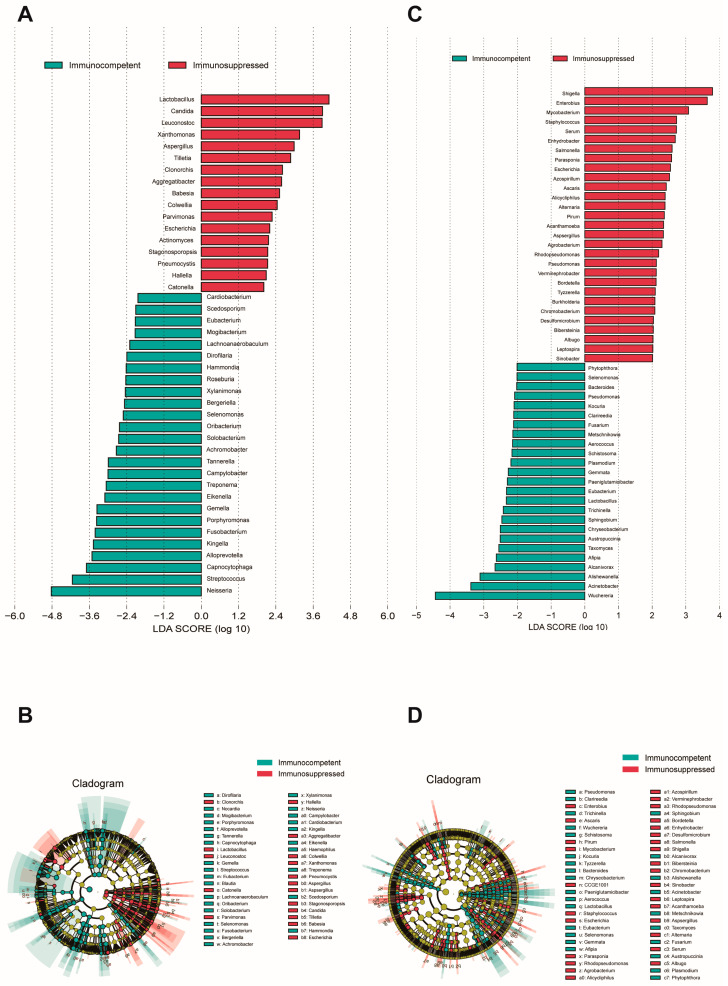
LEfSe analysis of microbial communities in sputum and BALF specimens. (**A**) LDA scores showing enriched taxa in immunocompetent (blue) and immunosuppressed (red) groups in sputum specimens. (**B**) Cladogram indicating the phylogenetic distribution of microbiota correlated with the two groups in sputum specimens. (**C**) LDA scores of enriched taxa between groups in BALF specimens. (**D**) Cladogram indicating the phylogenetic distribution of microbiota correlated with the two groups in sputum specimens. The LEfSe analysis included all taxa. Taxa with an LDA effect size value > 2 were described. Statistical significance was set at *p* < 0.05.

To identify species with a differential abundance between groups, an LEfSe analysis was performed on both cohorts’ specimens (LDA score > 2, *p* < 0.05). An analysis of sputum specimens revealed a significant enrichment of fungal species in the respiratory microenvironment of immunosuppressed patients (Figure 2A,B), where notable genera included *Candida*, *Aspergillus*, and *Cryptococcus*. Other predominant species were primarily associated with the oral microbiome, upper respiratory tract-related microbiota, viruses, and some rare plant pathogens. In sputum, the immunocompetent patients showed an enrichment of oral cavity-associated taxa and anaerobic bacteria, such as *Neisseria, Streptococcus, Porphyromonas,* and *Solobacterium,* which are typically considered part of the normal oral microbiome. In BALF specimens, a substantial enrichment in *Aspergillus* was similarly observed in the immunosuppressed group (Figure 2C,D). Additionally, genera typically associated with the gut microbiome, such as *Shigella, Salmonella,* and *Staphylococcus*, were found to be enriched in this cohort. BALF from immunocompetent patients showed an enrichment in *Bacteroides, Chryseobacterium*, and various environmental microbes that may represent a more balanced microbiome. Interestingly, we observed that *Aspergillus fumigatus*, *Aspergillus flavus*, and *Escherichia fergusonii* were consistently enriched in both specimen types from immunosuppressed patients.

### 3.4. Tighter Co-Occurrence Network Structure Associated with Immunosuppression

**Figure 3:**
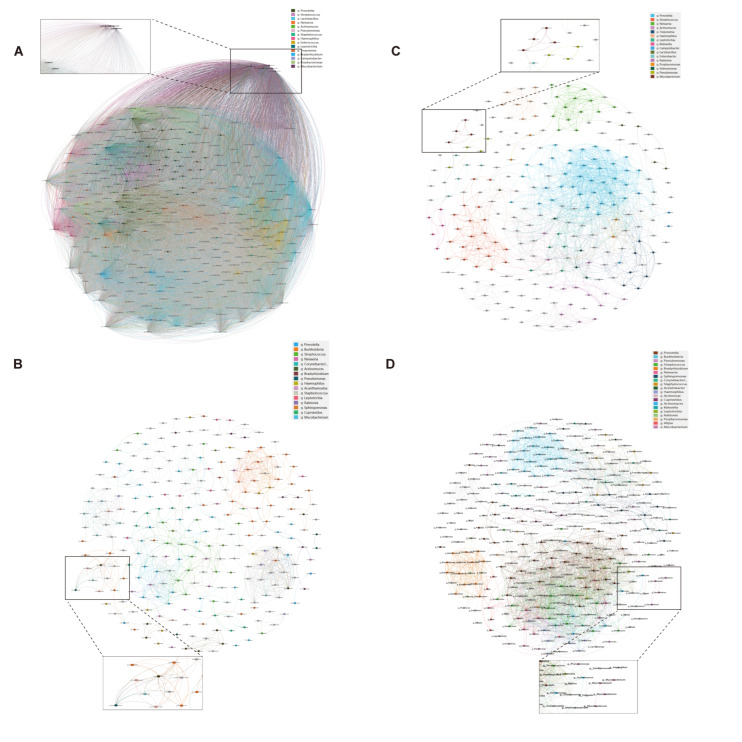
Microbial co-occurrence networks in sputum and BALF specimens. Bacterial co-occurrence networks in immunosuppressed (**A**,**B**) and immunocompetent (**C**,**D**) patients from sputum (**A**,**C**) and BALF (**B**,**D**) specimens. Nodes represent bacterial genera; node size indicates relative abundance. Edges show significant correlations between taxa (Spearman’s |ρ| > 0.4, *p* < 0.05). Colors indicate different bacterial modules. Insets show subnetworks centered on *Mycobacterium* genus. Networks were visualized using Gephi with ForceAtlas2 layout algorithm.

The co-occurrence network analysis revealed distinct microbial interaction patterns between immunosuppressed and immunocompetent groups (Table A1). The immunosuppressed group exhibited a significantly higher network complexity (Figure 3), with increased edges (BALF: 825 vs. 2122, *p* < 0.01; sputum: 62,449 vs. 1375, *p* < 0.001) and network density (BALF: 0.027 vs. 0.044, *p* < 0.05; sputum: 0.999 vs. 0.047, *p* < 0.001). Notably, the *Mycobacterium* genus showed enhanced connectivity in the immunosuppressed group, forming significant correlations with 157 taxa in sputum and 48 taxa in BALF (*p* < 0.001), compared to 23 and 12 correlations, respectively, in the immunocompetent group. This enhanced bacterial interaction, coupled with reduced modularity, suggests a restructured microbial community with potentially altered metabolic dependencies in immunosuppressed hosts.

### 3.5. Association Between Microbial Community and Host Immune Status

**Figure 4:**
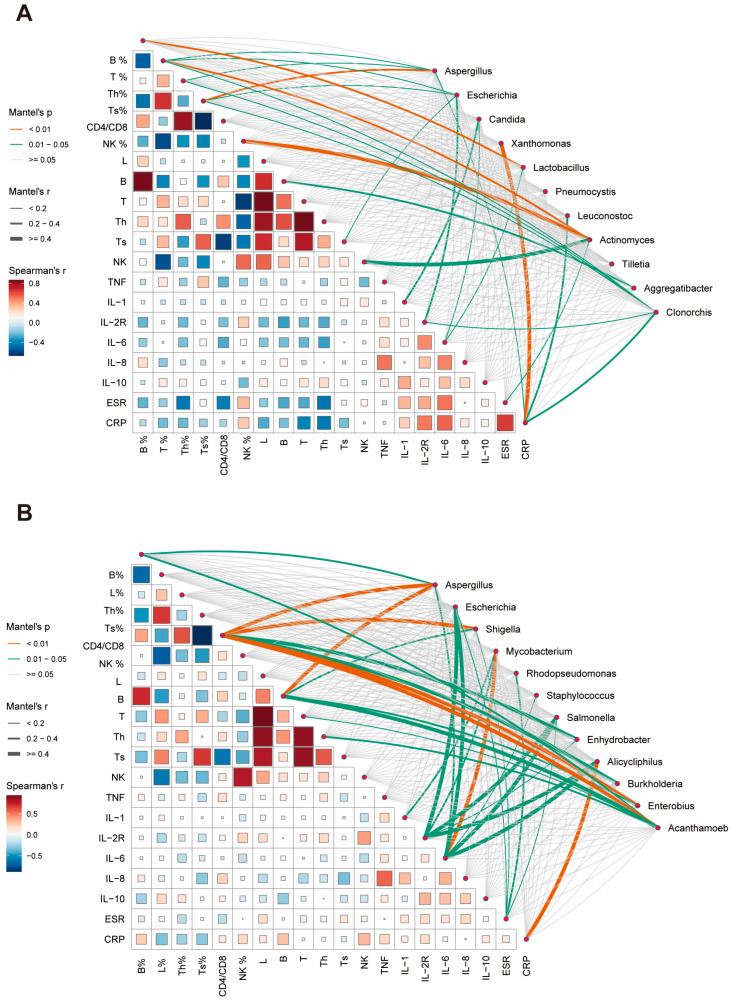
Correlation analysis between clinical parameters and microbial abundance in respiratory specimens. (**A**) Correlation network showing relationships between clinical indicators and microbial taxa in sputum specimens. Line thickness indicates Mantel’s r value, line color represents significance level (*p* < 0.01, orange; 0.01 ≤ *p* < 0.05, green). Square size and color intensity reflect Spearman’s correlation coefficient. (**B**) Corresponding correlation network for BALF specimens. Th: T helper cell; Ts: T suppressor cell; NK: natural killer cell; TNF: tumor necrosis factor; IL: interleukin; ESR: erythrocyte sedimentation rate; CRP: C-reactive protein.

We subsequently endeavored to evaluate potential relationships between the abundance changes in predominant species and patients’ clinical characteristics, aiming to elucidate their roles in host inflammatory or immune responses. In sputum specimens, B% was found to correlate with *Lactobacillus* abundance. T%, CD4%, and CD8% were influenced by multiple dominant species. *Actinomyces* demonstrated a strong association with NK-cells and NK%. *Candida* primarily correlated with inflammatory factor expression (Figure 4A). BALF specimens exhibited stronger overall correlations compared to sputum specimens. Immunological cell indicators were predominantly associated with *Aspergillus, Shigella*, and *Acanthamoeba*. Changes in inflammatory factors were largely correlated with species such as *Escherichia*, *Salmonella*, and *Alicycliphilus*. Notably, a strong correlation was observed between *Mycobacterium* and IL-6 (Figure 4B).

These immune-related indicators showed statistically significant differences between the immunosuppressed and control groups (Table 3), suggesting that these predominant species may directly or indirectly influence the immune status, as a result of which affecting the expression of relevant clinical immune or inflammation-related markers.

## 4. Discussion

This study aimed to analyze the differences in respiratory microbiota between immunosuppressed and immunocompetent NTM-PD patients using multiple specimen types. Our findings reveal that immunosuppressed patients exhibit a reduced microbial diversity and altered community composition in their pulmonary microbiome, a phenomenon consistently observed in both sputum and BALF specimens. Specimens from immunosuppressed patients demonstrated an enrichment in fungi and opportunistic pathogens, with a higher NTM burden and tighter community interactions in co-occurrence networks. The LEfSe analysis of sputum and BALF specimens identified common enriched species across the upper and lower respiratory tract, such as *Aspergillus fumigatus*, *Aspergillus flavus*, and *Escherichia fergusonii*. Compared to immunocompetent patients, immunosuppressed individuals showed lower clinical indicators including B%, T%, and NK%, all of which correlated with predominant species in the specimens. These results underscore the significant role of the respiratory microbiome in host inflammatory responses and immune regulation.

Previous studies by Charlson et al. [28] investigated respiratory microbiome alterations in lung transplant recipients, while Singh et al. [29] analyzed the impact of corticosteroid therapy on the respiratory microbiome, both revealing a significantly reduced microbial diversity in immunosuppressed states. Sulaiman et al. [12] also examined how NTM infections modify the pulmonary microenvironment in affected patients. However, research specifically examining microbiome characteristics across different immune states in patients with NTM pulmonary disease remains relatively scarce, which constitutes the innovative aspect of our present study.

In our cohort, immunosuppressed patients constituted approximately 25% of the total, consistent with the previous literature [30]. Baseline analysis revealed a higher proportion of females and increased COPD comorbidity, both of which may increase susceptibility to various pathogens [15,31]. Microbial diversity analysis revealed distinct patterns between sputum and BALF specimens. In sputum specimens, significant differences were observed in the Shannon index and PCoA (*p* < 0.05) but not in Chao1 index, suggesting an altered community composition and evenness but preserved species richness in immunosuppressed patients. Conversely, BALF specimens showed significant differences only in β-diversity (*p* < 0.01), with no changes in α-diversity metrics, indicating that immune status primarily affects the lower airway microbial community structure. These findings align with previous observations by Sulaiman et al. [12], suggesting that sputum cannot be used as a surrogate for the lower airways to study the airway microbiome.

An analysis of NTM abundance showed contrasting patterns across specimen types. BALF specimens exhibited a higher NTM relative abundance than sputum specimens, suggesting BALF may better reflect the actual NTM burden in the pulmonary microenvironment. Notably, immunosuppressed patients demonstrated a significantly lower NTM abundance in sputum (0.75% vs. 1.60%, *p* = 0.014) but a higher abundance in BALF (5.18% vs. 3.82%, *p* = 0.042) compared to immunocompetent individuals. This inverse pattern likely reflects impaired sputum expectoration in immunosuppressed patients, indicating BALF sampling may provide a more accurate assessment of pulmonary NTM colonization in this population [32]. Interestingly, our study found that NTM detection rates in sputum specimens were higher in immunocompetent individuals than in immunosuppressed patients, though this difference did not reach statistical significance. We hypothesize that this trend may be related to immunosuppressed patients’ inability to produce high-quality sputum samples due to diminished cough responses [33], as well as the normal pathogen-fighting immune processes in control patients that promote the release of NTM from intracellular to extracellular environments, facilitating their entry into sputum [34,35,36]. This observation further highlights the limitations of sputum specimens in accurately representing the respiratory microbiome, particularly in immunosuppressed populations.

The LEfSe analysis revealed an enrichment in multiple pathogens in the immunosuppressed group across both specimen types. Fungal dominance, particularly *Candida*, was more pronounced in sputum specimens, while bacterial dominance was more evident in BALF. The presence of viruses and plant pathogens likely relates to environmental exposure, as patients with compromised immune systems may fail to effectively clear these typically harmless environmental microorganisms from their respiratory tract, allowing for persistent colonization [37] BALF demonstrated an enrichment in gut-related bacteria. Previous research by Ferrer et al. [38] found that antibiotic treatment can eliminate susceptible microorganisms, thereby conferring ecological advantages to antibiotic-resistant *Shigella* and *Escherichia* strains. However, since their antibiotic history showed no significant difference between our two patient groups (*p* = 0.35), the presence of these typical gut microbiota members in the lungs may more strongly reflect gut–lung axis dysregulation. Dickson et al. [39] highlighted that intestinal microbiome abnormalities can directly influence the pulmonary microbiome composition and immune function. In immunosuppressed patients, an impaired intestinal barrier function allows gut microorganisms or their metabolic products to translocate to the lungs via systemic circulation. These differences reflect the distinct microbial composition at different sampling sites and reaffirm the limitations of sputum in representing lower respiratory tract microbiota [40].

The co-occurrence network analysis revealed markedly different characteristics between groups. The immunosuppressed group exhibited a highly connected, tightly clustered network, while the other one showed a relatively sparse network with richer community structures. The high connectivity in the immunosuppressed group may represent a closely associated community formed under abnormal immune conditions, where NTM-associated taxa may explain the frequent dysbiosis and poor response to antimicrobial therapy [41,42]. The diverse community structure in the immunocompetent group might represent a more balanced and resilient microbial ecosystem.

The altered microbiota in immunosuppressed patients may further suppress host immune responses against NTM. Multiple immune and inflammatory parameters were significantly dysregulated in these patients. The Mantel test revealed significant correlations between predominant taxa and these clinical indicators, suggesting potential interactions between microbial dysbiosis and immune dysfunction. Our study also demonstrated NTM enrichment in BALF and a strong correlation with IL-6, a finding not previously reported and possibly unique to immunosuppressed NTM-PD patients [12]. *Aspergillus* and *Escherichia* were enriched in both specimen types, suggesting potential synergistic roles in NTM invasion and colonization. Previous studies [43] have indicated a significant association between NTM-PD and *Aspergillus* infection, often resulting in more severe clinical manifestations and a poorer prognosis. Although the underlying mechanisms remain unclear, this co-infection pattern may be linked to pre-existing pulmonary conditions in immunosuppressed patients [44]. *Escherichia fergusonii*, an emerging opportunistic pathogen, has been increasingly associated with infections in immunosuppressed patients; however, its connection with NTM has not been reported [45]. Its similarity to *E. coli*, coupled with antibiotic resistance and biofilm formation capabilities, may contribute to treatment difficulties. We hypothesize that *E. fergusonii* colonization may play a crucial role in the poor prognosis observed in immunosuppressed NTM-PD patients.

This study has several limitations. In our baseline data, the immunosuppressed group had a significantly lower proportion of female patients compared to the immunocompetent group, whereas Dickson et al. [39] demonstrated that females typically exhibit higher pulmonary microbiome diversity than males. Additionally, the immunosuppressed cohort had a significantly higher prevalence of COPD. Previous research has suggested that the reduced muco-ciliary clearance in COPD patients could influence microbial colonization, while chronic inflammation also creates distinct microenvironmental differences. Furthermore, the immunosuppressed group exhibited significantly higher rates of pulmonary co-infection, which could alter the pulmonary microenvironment through various mechanisms, including microbial interactions, biofilm formation, and host immune modulation. Although we employed ANCOVA adjustments in subsequent analyses, these baseline differences might still have confounded our final conclusions.

When analyzing baseline data, we found no significant difference in antibiotic history between the two groups (*p* = 0.35). However, according to the previous literature, immunosuppressed patients typically demonstrate higher rates of antibiotic use. Henkle et al. [46] found that antibiotic usage rates were significantly higher in immunosuppressed patients compared to immunocompetent individuals. Similarly, Andréjak et al. [47] reported an elevated exposure to broad-spectrum antibiotics among immunosuppressed patients. Considering our study’s relatively small sample of immunosuppressed patients (*n* = 32), its single-center design, and the inclusion of newly diagnosed patients in the immunosuppressed cohort introducing selection bias, antibiotic use might still have represented an important confounding factor affecting microbial environmental differences between groups, despite the slightly higher but statistically non-significant antibiotic usage rate in the immunosuppressed group. Therefore, the groups were not homogeneous in terms of several characteristics, highlighting the need for future research with larger sample sizes and more detailed antibiotic usage categorization. BALF specimens were available for only a subset of patients, and the conclusions drawn from BALF did not entirely align with those from sputum specimens. This discrepancy warrants further investigation of core microbiota transmission from sputum to BALF in paired samples, particularly focusing on the role of shared bacterial communities in shaping the lower airway microenvironment. Furthermore, while we identified *Aspergillus* and *Escherichia* as predominant species involved in clinical indicator regulation in immunosuppressed patients, their regulatory patterns differed between specimen types. Larger specimen sizes are needed to determine whether these differences stem from BALF specimen size limitations or reflect genuine differences in microbial interaction patterns at different sites.

## 5. Conclusions

In conclusion, the metagenomic analysis of sputum and BALF revealed altered microbial diversity and community structures in immunosuppressed NTM-PD patients compared to immunocompetent controls. BALF specimens demonstrated superior sensitivity in detecting a pulmonary NTM burden, with immunosuppressed patients showing a significantly elevated NTM abundance. These patients exhibited more dynamic and interconnected microbial regulatory networks, characterized by an enrichment in predominant taxa that correlated significantly with immune and inflammatory parameters. Our findings suggest a dual therapeutic strategy: targeting the microbiome to modulate pulmonary bacterial communities and restrict mycobacterial colonization, while implementing host-directed immunotherapy. This approach provides novel perspectives for treating NTM-PD, particularly in immunosuppressed populations. The contribution of these microbial alterations to NTM-PD pathophysiology merits further investigation in larger cohorts.

## Figures and Tables

**Table 1:** Comparison of characteristics and clinical manifestations between immunocompetent and immunosuppressed patients with NTM infections.

	All Patient	Immunocompetent	Immunosuppressed	*p*-Value
Subjects	143	111	32	
Age years, mean ± SD	59.8 ± 13.5	57.9 ± 12.8	61.6 ± 14.1	0.17
Female	80 (56)	68 (61)	12 (38)	0.02
BMI, kg/m^2^	21.2 ± 3.8	21.4 ± 3.7	20.7 ± 4.1	0.344
Hypertension, *n* (%)	17 (12)	13 (12)	4 (13)	0.75
Diabetes	9 (6)	6 (5)	3 (9)	0.37
COPD	11 (8)	6 (5)	5 (16)	0.04
Bronchiectasis	51 (36)	43 (39)	8 (25)	0.21
Pulmonary co-infection	19 (13)	7 (6)	12 (38)	<0.01
Recent antibiotic use	44 (31)	32 (29)	12 (38)	0.35
HRCT thorax				
Cavities	27 (19)	20 (18)	7 (22)	0.61
Fibrosis	4 (3)	2 (2)	2 (6)	0.18
Bronchiectasis	65 (45)	52 (47)	13 (41)	0.57
Thickened airways	8 (6)	5 (5)	3 (9)	0.36
Nodules	40 (28)	31 (28)	9 (28)	1
Mucoid impaction	2 (1)	1 (1)	1 (3)	0.35
Specimen Type				
Sputum	111	79	32	
BALF	64	48	16	

**Table 2:** Comparison of *Mycobacterium* detection rates and relative abundance across specimen types and immune status.

Specimen Type	Cohort	Number of Specimens	Detection Rate (%)	*p*-Value	Relative Abundance (%, mean ± SD)	Fold Change	*p*-Value
Sputum	Immunosuppressed	32	65.63	0.59	0.75 ± 0.87	2.13	0.01
	Immunocompetent	79	70.89		1.60 ± 1.98		
BALF	Immunosuppressed	16	75.00	0.76	5.18 ± 1.99	0.74	0.04
	Immunocompetent	48	66.67		3.82 ± 2.96		

Fold change was calculated as the ratio of mean relative abundance between immunosuppressed and immunocompetent groups.

**Table 3:** Clinical characteristics and immune parameters of patients with NTM-PD by immune status.

	Immunocompetent *n* = 111	Immunosuppressed *n* = 32	*p*-Value
Lymphocyte percentage (*n* = 132) (%)			
B lymphocyte	13.8 ± 7.4	8.2 ± 5.9	0.002 **
T lymphocyte	69.2 ± 8.5	72.4 ± 14.8	0.234
CD4+ lymphocyte	41.2 ± 9.4	36.8 ± 14.3	0.035 *
CD8+ lymphocyte	24.1 ± 8.6	29.3 ± 13.2	0.041 *
CD4+/CD8+ T lymphocyte ratio	1.9 ± 0.8	1.4 ± 0.9	0.015 *
NK lymphocyte	16.4 ± 8.7	19.8 ± 13.2	0.168
Absolute count of lymphocyte subsets (IQR) (/μL)	1144.0 (859.0–1642.0)	1156.9 (704.0–1527.1)	0.862
B lymphocyte	151.0 (98.0–307.0)	94.0 (55.0–166.0)	0.024 *
T lymphocyte	785.0 (553.0–1107.0)	619.0 (553.0–841.0)	0.186
CD4+ lymphocyte	478.0 (344.0–593.0)	417.0 (272.0–424.0)	0.042 *
CD8+ lymphocyte	299.0 (186.0–431.0)	309.0 (115.0–438.0)	0.984
NK lymphocyte	177.0 (130.0–244.0)	187.0 (83.0–363.0)	0.862
Inflammatory factors (IQR) pg/mL			
TNF-α	8.2 (5.3–16.8)	9.7 (5.9–19.2)	0.286
IL-1	5.0 (5.0–5.9)	5.0 (5.0–11.7)	0.142
IL-2R	519.5 (381.0–751.0)	738.0 (608.0–1192.0)	<0.001 ***
IL-6	4.2 (2.6–7.9)	16.9 (6.7–51.4)	<0.001 ***
IL-8	12.0 (9.0–25.0)	14.0 (12.0–35.0)	0.124
IL-10	5.0 (5.0–5.0)	5.0 (5.0–5.0)	0.856
ESR (mm/h)	25.0 (13.0–53.0)	49.0 (19.0–98.0)	0.008 ***
CRP (mg/L)	3.2 (1.7–16.4)	21.2 (7.0–119.0)	<0.001 ***

Abbreviations: TNF: tumor necrosis factor; IL: interleukin; ESR: erythrocyte sedimentation rate; CRP: C-reactive protein * *p* < 0.05, ** *p* < 0.01, *** *p* < 0.001.

## Data Availability

The data presented in this study are available on request from the corresponding author.

## Abbreviations

The following abbreviations are used in this manuscript:

NTM-PD	Nontuberculous mycobacterial pulmonary disease
BALF	Bronchoalveolar lavage fluid
PCoA	Principal Coordinates Analysis
LEfSe	Linear discriminant analysis Effect Size
NTM	Nontuberculosis Mycobacterium
NGS	Next-Generation Sequencing
ANCOVA	Analysis of Covariance
BH	Benjamini–Hochberg
COPD	Chronic Obstructive Pulmonary Disease

## Appendix A

**Table A1:** Network properties of microbial co-occurrence in NTM-PD patients.

Parameters	Immunosuppressed	Immunocompetent	*p*-Value
BALF specimens			
Nodes	250	311	0.028 *
Edges	825	2122	<0.001 ***
Positive correlations	824	2122	<0.001 ***
Negative correlations	1	0	0.317
Average degree	6.60	13.65	<0.001 ***
Average path length	2.89	2.83	0.245
Network diameter	8.06	9.51	0.032 *
Network density	0.027	0.044	0.015 *
Clustering coefficient	0.791	0.476	<0.001 ***
Sputum specimens			
Nodes	354	243	0.008 **
Edges	62449	1375	<0.001 ***
Positive correlations	41403	1375	<0.001 ***
Negative correlations	21046	0	<0.001 ***
Average degree	352.82	11.32	<0.001 ***
Average path length	0.007	1.77	<0.001 ***
Network diameter	0.018	4.49	<0.001 ***

Nodes represent individual bacterial taxa; edges represent significant correlations (Spearman’s |ρ| > 0.4, *p* < 0.05) between taxa. Statistical comparisons were performed using permutation tests with 1000 randomizations. * *p* < 0.05, ** *p* < 0.01, *** *p* < 0.001.

The species-level characterization of NTM isolates revealed distinct distribution patterns across different respiratory specimens and immune status (Figure A1). In sputum samples from immunocompetent patients (A), *M. intracellulare* predominated (48%), followed by *M. abscessus* (28%), with *M. avium* and other species comprising smaller proportions. Sputum from immunosuppressed patients (B) maintained a similar species distribution, though with a slightly increased *M. intracellulare* prevalence (51%). In BALF specimens, immunocompetent patients (C) demonstrated a comparable distribution to their sputum counterparts, whereas immunosuppressed patients (D) showed a notable shift toward an increased *M. abscessus* representation (39%) with a proportionally decreased *M. intracellulare* (48%) compared to other groups. This distinctive species distribution pattern in BALF from immunosuppressed patients suggests potential compartment-specific ecological adaptations influenced by immune status.
